# Damage to Hospitals by Hostile Air-Craft

**Published:** 1915-01-30

**Authors:** Charles Larking

**Affiliations:** Chairman of the Board of Management. Norfolk and Norwich Hospital, Norwich


### THE EDITOR'S LETTER-BOX.

### Damage to Hospitals by Hostile Air-Craft.

We have received from Mr. Charles Larking, the chairman of the board of management of the Norfolk and Norwich Hospital, a copy of a letter which he wrote to the Prime Minister asking him to give the board of management an assurance that any damage done by the enemy to the Norfolk and Norwich Hospital, and to their convalescent home at Cromer, would be taken in hand by the Government without delay. We print the letter of Mr. Larking to the Prime Minister and the reply as sent to him by the Treasury, from which it appears that the Government will not give any general assurance that relief will be given from public funds in respect of damage incurred through the action of the enemy. This reply is not encouraging, but we have no doubt that, should occasion arise and the hospitals combine to bring the actual facts to the notice of the authorities, such a course is not likely to fail to secure justice for the voluntary hospitals from the Government, as the circumstances of each case may seem to warrant.

**To the Prime Minister, Downing Street, London.**

Sir,—Adverting to the official announcement that the Government has resolved to provide relief from Imperial funds "in respect to damage to persons and property sustained in the recent bombardment of the towns of Scarborough, Whitby, and the Hartlepools," I am directed by my board of management to write and ask you whether the Government intends to treat as a national liability any such damage done by the enemy in future.

I may remind you that this hospital has already received 900 wounded soldiers from the Front, and of course hopes to continue this beneficent work as the war proceeds.

It would, therefore, be a national disaster if any portion of this hospital, or its convalescent home at Cromer, were damaged by air-craft or bombardment, without prospect of prompt restoration; and the annual voluntary contributions are quite inadequate to meet such a contingency. Further, the future usefulness of these institutions would be seriously impaired by such a disaster if additional financial aid were not forthcoming.

As you are aware, the premium for insuring against this risk is now prohibitive; consequently my board of management would feel greatly relieved by an assurance that any damage done to their property would be taken in hand by the Government without delay.—I remain, Sir, your obedient servant,

Charles Larking,
Chairman of the Board of Management.

Norfolk and Norwich Hospital, Norwich,
January 2, 1915.

